# Microstructural and histochemical analysis of aboveground organs of *Centaurea cyanus* used in herbal medicine

**DOI:** 10.1007/s00709-019-01437-4

**Published:** 2019-09-12

**Authors:** Weronika Haratym, Elżbieta Weryszko-Chmielewska, Agata Konarska

**Affiliations:** Department of Botany, Faculty of Horticulture and Landscape Architecture, University of Life Sciences in Lublin, Akademicka 15, 20-950 Lublin, Poland

**Keywords:** Micromorphology and anatomy, *Centaurea*, Scanning electron microscopy, Histochemistry and ultrastructure, Secretory structures, Medicinal plant

## Abstract

*Centaurea cyanus* L. is a valuable source of many different bioactive substances. It is used in herbal medicine, but the structure of its organs used as raw material and secretory tissues has been insufficiently examined. The aim of this paper was to investigate the microstructure of *C. cyanus* flowers, bracts, stems and leaves with particular emphasis on secretory structures. Moreover, the main classes of secondary metabolites present in the secretion were identified and the taxonomic value of some micromorphological and anatomical features was analysed. Histochemical, micromorphological and ultrastructural analyses of aboveground organs of *C. cyanus* were carried out using light, fluorescence, scanning and transmission electron microscopy. The analyses revealed the presence of petal papillae and a characteristic cuticular pattern on the petals, stamens and stylar hairs. There were four types of non-glandular trichomes on the bracts, leaves and stem surfaces. The epidermal cells of the bracts contained prismatic calcium oxalate crystals. Two kinds of secretory structures, i.e. glandular trichomes and ducts, were observed in the *C. cyanus* organs. The glandular trichomes were located on the bract and stem surfaces, and the ducts were detected in the leaves and stems. Ultrastructural analyses of the epithelium of the ducts showed the presence of strongly osmiophilic insoluble phenolic material in vacuoles as well as moderately osmiophilic insoluble lipidic material in elaioplasts and vesicles. The results of histochemical assays showed a heterogeneous nature of the duct secretion, which contained essential oil, lipids, flavonoids, tannins and terpenes containing steroids.

## Introduction

*Centaurea* L. is a large polymorphous genus from the tribe Cardueae (Asteraceae family). It comprises annual, biennial and perennial grassy plants occurring worldwide, especially in America, Europe, North Africa and Asia (Wagenitz and Hellwig 1996; Susanna and Garcia-Jacas 2009; Rai and Kon 2013). Only 21 species grow in Poland. They are mostly associated with *Festuco-Brometea* and anthropogenic communities (Mirek et al. 2002).

The best-known species is *Centaurea cyanus* L*.*, which originated in the Caucasus. Since ancient times, it has dispersed as a weed into crop fields, especially in wheat and canola plantations, or along field margins (Rösch 1998; Wassmuth et al. 2009; Boršic et al. 2011). This annual plant has a branched stem and a tap root system. Its lanceolate leaves are arranged alternately on the stem. Flowers appear from the first week of June to mid-August, reaching full bloom in the first week of July. The plant produces inflorescences composed of sterile peripheral deep blue ray florets and fertile tubular disc florets with an ovary containing a single ovule (Denisow 2006). The fruit is an achene (Chiru et al. 2013).

The medicinal raw material *Cyani flos* is used in herbal medicine (Polish Pharmacopoeia VIII 2008). The main active ingredients of *cyani flos* are amino acids, aromatic acids, coumarins, flavonoid derivatives, indole alkaloids, phenylcarboxylic acids, polyphenols and polysaccharides (Litvinenko and Bubenchikova 2007; Muravéva and Bubenchikova 2007; Pirvu et al. 2015). With its content of these substances, this plant is reported to have anti-inflammatory, antimicrobial, antipruritic, antitussive, astringent, cholagogic, diuretic, emmenagogue, gastroprotective, immunological, ophthalmic, purgative and many other biological activities (Garbacki et al. 1999; Senderski 2007; Chiru 2009; Pirvu et al. 2012). Medicinal properties have also been detected in seeds, which are used as mild laxatives; leaves used for production of cleansing facial spray and decoction with antirheumatic activity; and stems, which exhibit antibacterial activity (Garbacki et al. 1999; Stanojković et al. 2008; Pirvu et al. 2015).

Micromorphological traits of the structure of Asteraceae flowers are important clues for classification of the family. They can be taxonomic markers for genera and tribes (Angulo and Dematteis 2014). The authors have identified structures responsible for corolla pubescence in species of the genus *Lessingianthus* (Vernonieae), i.e. papillae and four trichome types. In almost all species, papillae were concentrated at the corolla lobe tips and trichomes were located on the adaxial surface at the apex of the corolla lobes. The occurrence of papillae only in the apical zone of corolla lobes and the absence of trichomes on the corolla were observed by Haratym and Weryszko–Chmielewska (2012). However, biseriate glandular trichomes were detected on corolla lobe tips in *Helichrysum* (Ascensão et al. 2001), whereas biseriate trichomes were observed on different corolla parts in *Chamomilla* and *Inula* (Sulborska 2011, 2013).

The cuticular patterns on the surface of ray flower petals were analysed in different tribes of Asteraceae. A crested pattern was found in all species from the tribe Mutisieae. However, no consistent cuticular patterns were described in other tribes (Baagøe 1977, 1978; Hansen 1991).

Different forms (lanceolate, ovate, ovate-lanceolate, linear) and sizes of anther apical appendages, different shapes (sagittate and cuneate) of the anther base, and the absence or presence of a style basal node were also distinguished as taxonomic features in *Lessingianthus* (Angulo and Dematteis 2014).

The stylar trichomes found in Asteraceae flowers play a role in secondary pollen presentation (Leins and Erbar 2006). A new micromorphological feature, i.e. cuticular patterns on stylar hairs, was observed in members of all tribes (44) of the Asteraceae family (Erbar and Leins 2015). The authors of those studies reported five types of cuticular striation: crested, triple, double, longitudinal and transverse patterns. Such cuticular patterns could be helpful in phylogenetic classification.

The distinctive features of plants from the Asteraceae family include the presence of secretory canals and different types of glandular and non-glandular trichomes, which have high taxonomic value. The number and distribution of secretory canals in the stem, which are usually lined with epithelium, are helpful in identification of genera (Metcalfe and Chalk 1972). Secretory canals have been observed in stems and leaves in many genera, inter alia *Arnica* (Kromer et al. 2016), *Centaurea* (Chiru et al. 2013), *Inula* (Sulborska 2007), *Matricaria* (Andreucci et al. 2008), *Petasites* (Haratym and Weryszko-Chmielewska 2012) and *Rhaponticum* (Łotocka and Geszprych 2004).

Glandular trichomes are widely distributed throughout the family Asteraceae (Metcalfe and Chalk 1972). Their structure may be (i) uniseriate: *Aldama* and *Helianthus* (Aschenbrenner et al. 2013; da Silva et al. 2014), (ii) biseriate: *Chamomilla* (Andreucci et al. 2008; Sulborska 2011), *Helichrysum* (Ascensao et al. 2001), *Helianthus* (Göpfert et al. 2010), *Inula* (Sulborska 2013), *Stevia* (Bondarev et al. 2010) and (iii) multiseriate, as those described in *Sigesbeckia* (Heinrich et al. 2002) and in *Tussilago*, which had a long biseriate stalk and a multicellular head (Muravnik et al. 2016).

Non-glandular trichomes in Asteraceae exhibit a wide range of structure types. Metcalfe and Chalk (1972) listed nine types of hairs in this group; in *Centaurea*, they described uniseriate trichomes with a long terminal cell. Furthermore, as reported by Chiru et al. (2013), *Centaurea cyanus* has several types of non-glandular (protective) trichomes. With its white cobweb-like pubescence consisting of non-glandular trichomes covering the leaves and stem, the plant looks dull and grey (Rzymowska and Skrzyczyńska 2007; Chiru et al. 2013).

Although many studies have described the medicinal properties of different species from the *Centaurea* genus, the microstructure of their organs used as herbal raw material and secretory tissues has been rarely investigated. Some information provided by microscopic analyses of the vegetal product *Cyani herba* was only published by Chiru et al. (2013). Given the importance of this species as a medicinal plant, the present study consisted in micromorphological, ultrastructural and histochemical analyses of aboveground organs of *C. cyanus* and their secretory structures, i.e. glandular trichomes and ducts, which are the main source of bioactive substances. Moreover, we considered the taxonomic value of some features of the floral micromorphology, secretory structures, non-glandular trichomes and calcium oxalate crystals, which may be useful for evaluation of the quality (falsification) of medicinal raw materials.

## Material and methods

The aboveground organs of *Centaurea cyanus* were examined at the full flowering stage in June and July 2014–2016. The plant material was obtained from the Botanical Garden of Maria Curie-Skłodowska University of Lublin, Poland (51° 15.629′ N and 22° 30.975′ E).

Flowers, bracts, stems (the middle part of the 6th and 7th internode) and leaves (from the 7th internode) were observed with the use of stereoscopic (SM), light (LM), fluorescence (FM), scanning electron (SEM) and transmission electron (TEM) microscopy. Moreover, various histochemical tests were performed on transverse sections of stems.

### SM and LM

Preliminary examination of fresh material (flowers, bracts, stems and leaves) was performed using a stereoscopic microscope equipped with a Nikon Coolpix 4500 camera and a Nikon Eclipse 400 light microscope.

At the floral stage, pollen grains were collected from the anther tube, spread on a slide, and stained with basic fuchsin. The size of the pollen grains (*n* = 100) was measured with a light microscope equipped with a calibrated ocular micrometer.

### Histochemical tests and FM

Transverse sections for these analyses were taken from the middle area between the 6th and 7th internodes of freshly collected stems. Only substances produced by secretory ducts were analysed. Fresh unfixed and unstained sections were used as a negative control. The classes of the tested metabolites are listed in Table 1. The observations were carried out with the use of a Nikon Eclipse 400 light microscope. Secondary fluorescence of plant metabolites was examined using antimony trichloride, magnesium acetate and aluminium trichloride under a Cy5 filter (excitation light—590–650 nm and a barrier filter—wavelength 663–738 nm). The autofluorescence of lipids was demonstrated using a fluorescein isothiocyanate-FITC filter (excitation light—590–650 nm and a barrier filter—wavelength 663–738 nm) (Huang et al. 2008). Images were obtained with the use of a digital camera Nikon Fi1 and NIS - Elements Br 2 software.

**Table 1:** Metabolite classes, reagents, reaction colours and references of the methodologies used in the histochemical and fluorescence tests

Metabolite classes	Reagents	Reaction colours	References
Lipids			
Total	Sudan IV	Orange	Johansen 1940; Lison 1960 Brundrett et al. 1991; Hohmann et al. 2001
	Sudan Red B	Red	
	Autofluorescence (FITC)	Green	Huang et al. 2008
Neutral and acidic lipids	Nile Blue	Blue	Jensen 1962
Terpenoids			
Essential oil and resin-oil	Nadi reagent	Purple	David and Carde 1964
Essential oil	Neutral Red	Red	Kirk 1970; Clark 1981
Steroids	Antimony trichloride	Yellow (under UV)	Mace et al. 1974
Sesquiterpenes	Conc. sulphuric acid	Yellow	Cappelletti et al. 1986
Phenolic compounds			
General	Ferric trichloride III	Black	Johansen 1940
Tannins	Potassium dichromate	Brown	Gabe 1968
Flavonoids	Aluminium trichloride	Yellow (under UV)	Charrière-Ladreix 1976
Flavonoids	Magnesium acetate	Yellow (under UV)	Charrière-Ladreix 1976
Polysaccharides			
General	PAS (periodic acid-Schiff’s) reagent	Pink	Mcmanus 1948
Pectins	Ruthenium Red	Crimson	Johansen 1940

### SEM

Small segments of stems, bracts and flowers (*n* = 10) were fixed in a 4% glutaraldehyde solution in 0.1 M phosphate buffer (pH 7.0). Subsequently, the samples were incubated for 12 h at room temperature. To wash the plant material, the same buffer was used four times at 20-min intervals. The samples were dehydrated in ethanol series (30, 50, 70, 90 and 95%) and submerged in absolute alcohol three times. After transferring to acetone, the plant material samples were critical point dried in liquid CO_2_ using Bal-Tec CPD 030. The prepared fragments of plant organs were placed on a double-sided carbon tape on stubs. A Polaron SC 7640 sputter coater was used for covering the specimens with a 10-μm-thick gold layer. A scanning electron microscope TESCAN/VEGA LMU at an accelerating voltage of 30 kV was used to examine the material.

### TEM

Small sections (5 × 5 mm) (*n* = 10) of *C. cyanus* stems were isolated and fixed in a mixture of 3.5% glutaraldehyde and 3.5% paraformaldehyde in 0.1 M phosphate buffer (PBS) with pH 7.2 for 1 h at room temperature. Subsequently, the specimens were rinsed in 0.1 M PBS and additionally fixed in a 4% aqueous solution of osmium tetroxide for 24 h at ambient temperature. Then, the samples were rinsed in distilled water, dehydrated with a graded ethanol series, and saturated in 1:3, 1:1 and 3:1 mixtures of LR White resin and acetone for 3 h each. Eventually, the prepared specimens were embedded in LR White resin and cut into ultra-thin sections of 60 to 90 nm using the Reichert Ultracut S microtome. The sections were loaded onto 100-mesh copper grids coated with Formvar (1% in ethylene dichloride) and stained with uranyl acetate and lead citrate (Reynolds 1963; Karnovsky 1965). The ultrastructure was analysed with the use of a Tesla BS 500 transmission electron microscope, and the viewed images were photographed on a Foton TN-12 electron microscope film.

## Results

### Morphology and anatomy of flowers

**Fig. 1:**
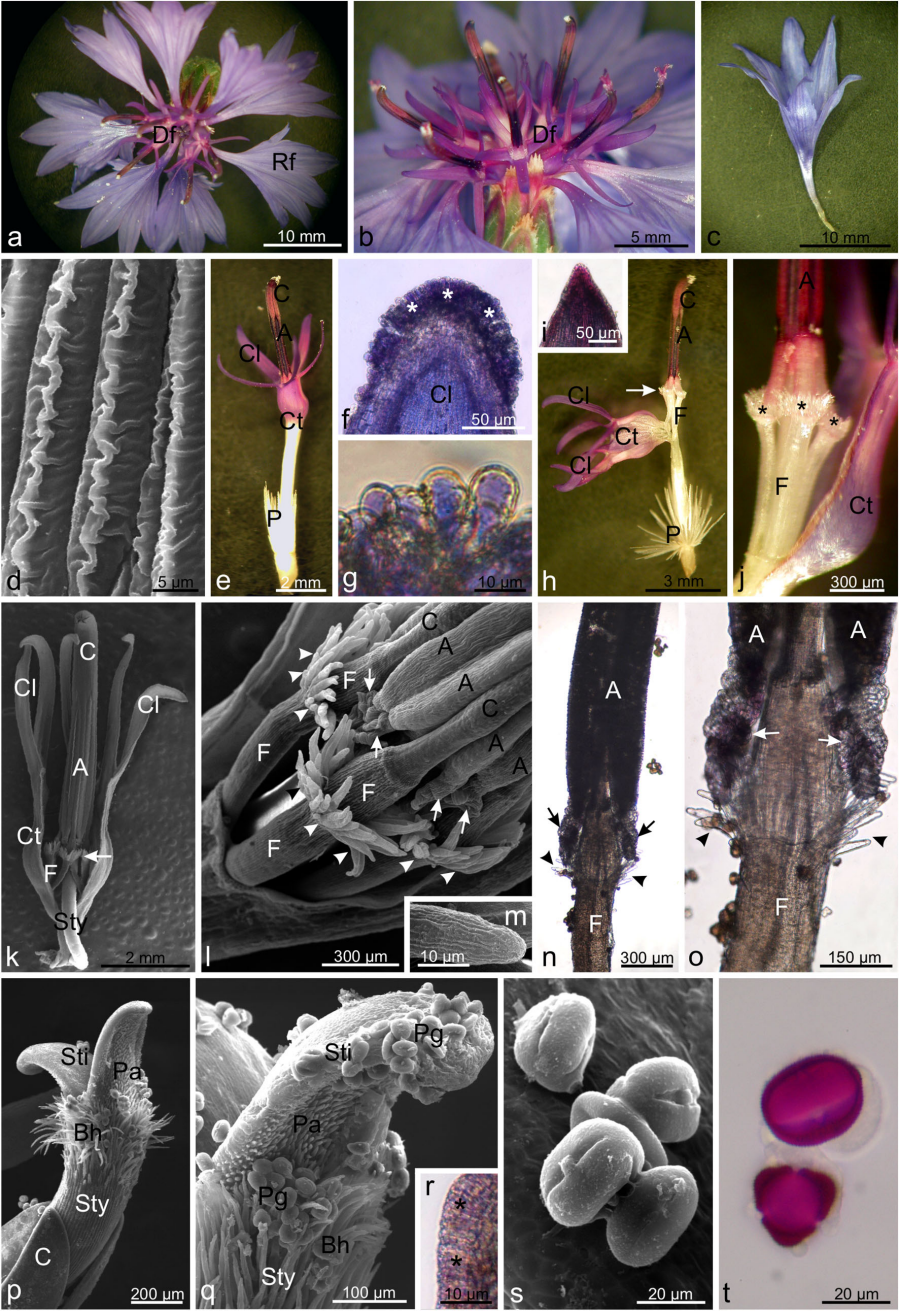
Characteristics of *Centaurea cyanus* flowers. **a** Inflorescence with marginal ray florets and central disc florets **b** Disc florets. **c** Ray floret. **d** Surface of the ray floret corolla with crested cuticular striae. **e** Disc floret. **f** Apical part of disc floret corolla lobes with papillae (*asterisks*) stained with anthocyanins. **g** Papillae on the corolla lobes. **h** Disc floret androecium with visible connectives, filaments and hair-like structures (*arrows*). **i** Apical part of a connective stained with anthocyanins. **j** Part of the androecium with filaments and hair-like structures (*asterisks*) in the form of a crown. **k** Transverse section through a disc floret with a visible androecium and hair-like structures (*arrows*). **l** Hair-like structures (*arrowheads*) on the filaments and spiral structures (*arrows*) on the basal part of the anthers. **m** Surface of a hair-like structure with longitudinal cuticular striae. **n**, **o** Parts of the stamen with hair-like (*arrowheads*) and spiral (*arrows*) structures. **p**, **q** Parts of the pistil with brushing hairs on the style and papillae on the stigma. **r** Section across elongated stigma epidermal cells stained with anthocyanins (*asterisks*) without papillae. **s** Tricolporate pollen grains. **t** Pollen grains stained with basic fuchsin in different views: equatorial view (*upper*) and polar view (*lower*). *Rf* ray flower, *Df* disc flower, *P* pappus, *Ct* corolla tube, *Cl* corolla lobes, *Sta* stamens, *C* connective, *A* anthers, *F* filaments, *Sti* stigma, Sty style, *Bh* style brushing hairs, *Pa* papillae, *Pg* pollen grains

The *Centaurea cyanus* was found to have a monopodial inflorescence. The flowers formed a single flower-like capitulum surrounded by involucral bracts (Fig. 1a). The peripheral florets were sterile, ligulate and funnel-shaped, and their colour ranged from blue through light violet to dark violet (Fig. 1a–c). Their epidermis was composed of elongated cells with distinctive cuticular striae forming a characteristic crested pattern on their surface (Fig. 1d). The inner part of the inflorescence was formed by disc florets (Fig. 1a, b, e). They were composed of five fused petals forming a tubular corolla (Fig. 1e), with adaxial epidermis at the top of the lobes covered with densely distributed approximately 27-μm-long papillae forming a violet-blue protuberance resembling a hemispherical surface (Fig. 1f, g). As in other epidermal cells, the vacuoles of the papillae contained anthocyanins. The anthers of five stamens formed a tube, which protruded from the corolla tube (Fig. 1e, h, k). The anther tube was approximately 47 mm long, and almost 30% of its length consisted of flattened apical parts of pink-violet connectives (Fig. 1e, h, i, k). Spirally twisted multicellular structures fused to the lower parts of the anthers (Fig. 1l n, o). Additionally, there were five separated white filaments (Fig. 1h–l). Below the top of the filaments, there were hair-like structures with longitudinal cuticular striae forming a crown (Fig. 1 l, m). These hairs contained cytoplasm with numerous granularities and cell nuclei (not shown). The upper part of the style and the outer part of the dichotomous stigma formed a secondary pollen presenter (Fig. 1p, q). Stylar hairs and numerous papillae were located on the surface of the presenter. The area of the style below the tip was covered by long brushing hairs and the tip and abaxial surface of the stigma were occupied by smaller and shorter papillae (Fig. 1p, q). The papillae were expanded at the base and pointed at the upper part. The stylar hairs were covered by longitudinal cuticular striae, similarly to the filament hairs (not shown). During maturation of the flower, the presenter presses through the centre of the anther tube and sweeps out the pollen released from the anthers. Retention of pollen grains is facilitated by the oblique orientation of the trichomes to the main axis of the style. The length of the part of the style with the presenter trichomes was 0.56–0.73 mm (on average 0.66 mm), which is about 5% of the entire length of the style. The adaxial epidermis of the stigma was composed of elongated cells, among which we did not find papillae (Fig. 1r). The stigma cells and the anther tube were pink-violet, which indicated the presence of anthocyanins.

The pollen grains of the *C. cyanus* are tricolporate, isopolar and radially symmetric (Fig. 1q, s, t). They are subtriangular in the polar view and compressed oval in the equatorial view. The length of the polar axis (P) ranges from 30.48 to 41.94 μm (on average 35.03 μm) and the equatorial axis (E) ranges from 24.13 to 30.48 μm (on average 27.39 μm). They represent medium-sized grains. Based on the ratio of the length of the polar axis to the equatorial diameter (P/E), which is on average 1.28, the pollen grains in the species are classified into the subprolate pollen type.

**Fig. 2:**
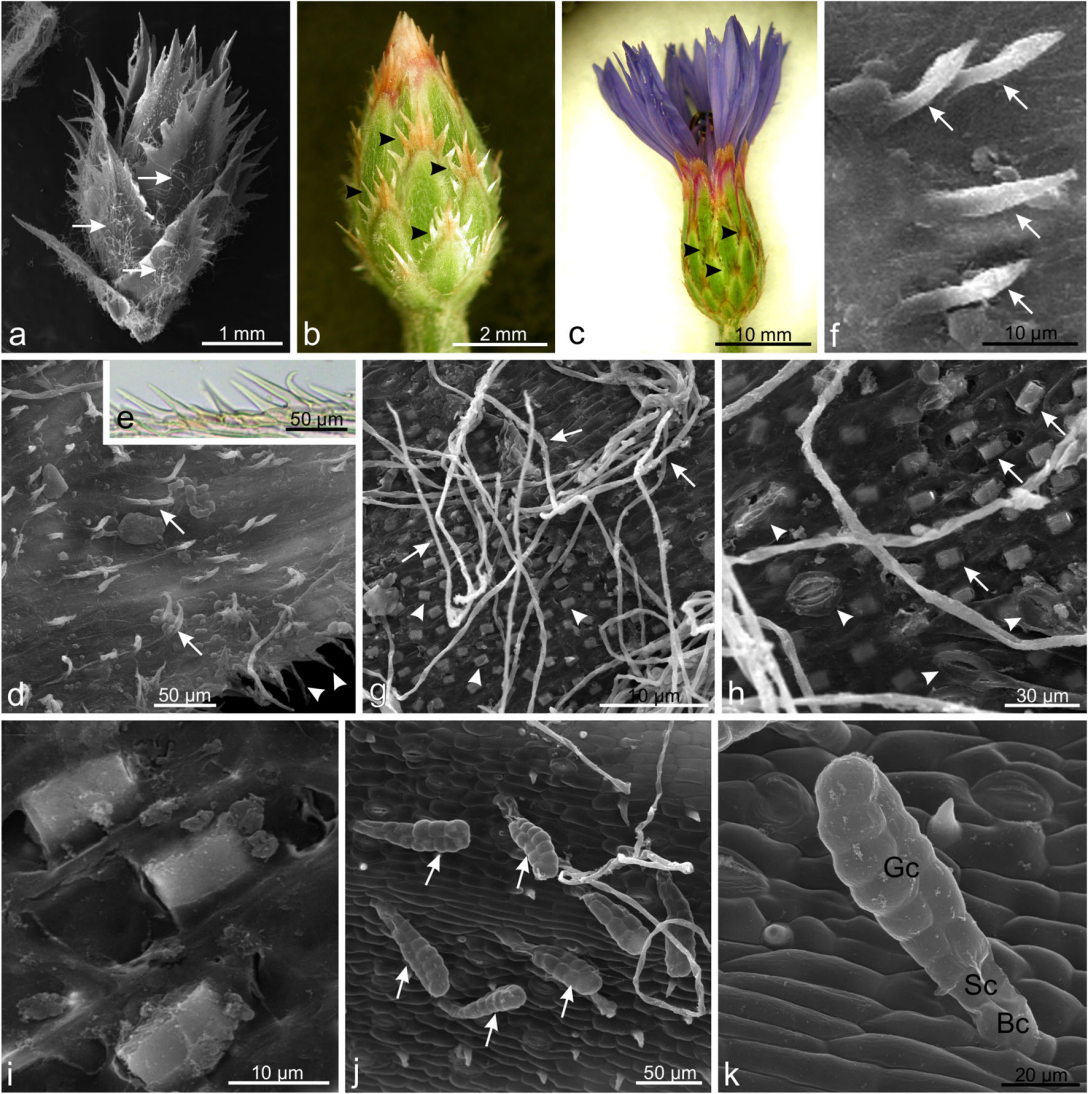
Microstructure of *C. cyanus* bracts. **a**, **b** Bracts on the floral buds with visible non-glandular trichomes (*arrows*) forming an indumentum and pointed white and beige trichomes at the edges (*arrowheads*). **c** Bracts during anthesis with brown coloured trichomes (*arrowheads*) on the edges. **d** Abaxial surface of the apical part of the bract with short unicellular pointed trichomes (arrows). **e** Portion of bract edges with pointed (*double-arrows*) and hook-shaped (*arrow*) trichomes. **f** Non-glandular, hook-shaped trichomes (*arrows*) on the abaxial surface of the bract. **g** Surface of the central abaxial part of the bract with long dead non-glandular trichomes (*arrows*) and calcium oxalate crystals (*arrowheads*). **h** Calcium oxalate crystals (*arrows*) and stomata (*arrowheads*). **i** Calcium oxalate crystals. **j** Glandular trichomes (*arrows*) on the abaxial bract surface. **k** Biseriate glandular trichome with a pair of basal cells (bc), a pair of stalk cells (sc), and 6 to 7 vertical tiers of glandular cells (gc)

The *C. cyanus* inflorescences subtended an involucre of overlapping bracts with serrate margins and toothed tips (Fig. 2a–c). During development of the inflorescence, the edged parts of the bracts changed colour from the base to the apex. Their visible portion became dark brown tanned (Fig. 2b, c). On the edges of each tooth of the bract, there were numerous pointed unicellular non-glandular trichomes (Fig. 2d, e). The abaxial surface of the bract was covered by two types of non-glandular trichomes. One type was represented by short hook-shaped unicellular hairs (Fig. 2d, f). Their sharp ends pointed towards the apex of the bract. Another type comprised long and tangled white multicellular hairs (Fig. 2g, h).

A few (7–10) glandular trichomes were also found in the central part of the abaxial surface of the bracts (Fig. 2j, k). They had a characteristic bilayered structure and consisted of a pair of basal cells, a pair of stalk cells, and 6 to 7 vertical tiers of glandular cells (Fig. 2j, k). The length of the trichomes was around 90 μm. During the observations, no pore or crack through which the secretion could be exuded was noticed.

Single calcium oxalate crystals were visible in the abaxial epidermal cells of the central part of the bract (Fig. 2g–i). These deposits represented the prismatic type and were approx. 10 μm in length and 5 μm in width. Moreover, numerous stomata were found in this epidermis of the bracts (Fig. 2h).

### Morphology and anatomy of stems

**Fig. 3:**
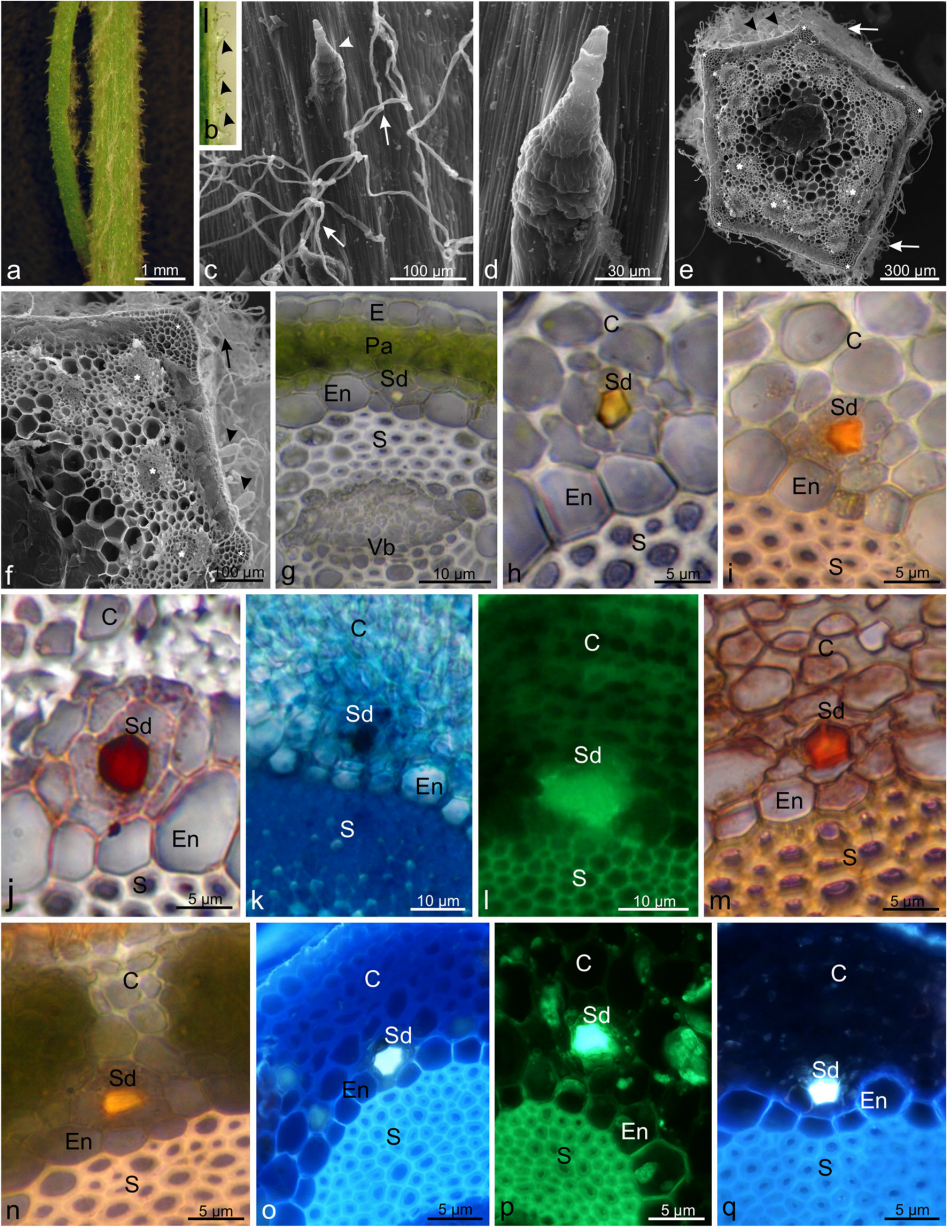
Characteristics of *C. cyanus* stems and presence of secondary metabolites in the secretory ducts. **a** Portion of a *C. cyanus* stem covered with trichomes. **b** Short non-glandular trichomes (*arrowheads*) on the stem. Scale bar = 250 μm. **c** Surface of the stem with long non-glandular trichomes forming a densely pubescent indumentum (*arrows*) and short pyramidal trichomes (*arrowhead*). **d** Short trichome on the stem surface. **e, f** Transverse sections through the stem with visible vascular bundles (*asterisks*), collenchyma in the corners (*stars*), and non-glandular (*arrows*) and biseriate glandular (*arrowheads*) trichomes on the surface. **g, h** Portion of a transverse section through the stem with a visible secretory duct with yellow secretion. **i** Lipophilic compounds in the secretion of the secretory duct stained orange with Sudan IV. **j** Lipids in the secretory duct stained red with Sudan Red B. **k** Acid lipids in the secretory duct stained dark blue after application of Nile Blue **l** Green autofluorescence under FITC indicating the presence of lipid substances. **m** Red coloured essential oil after treatment with Neutral Red. **n** Yellow-brown colour of tannins stained with potassium dichromate. **o** Secondary fluorescence of flavonoids observed after application of aluminium trichloride under the Cy5 filter. **p** Yellowish fluorescence of flavonoids observed addition of magnesium acetate under the Cy5 filter. **q** Yellowish fluorescence of steroids stained with antimony trichloride and observed under the Cy5 filter. *Sd* secretory ducts, *E* epidermis, *S* sclerenchyma, *C* collenchyma, *Pa* cortex parenchyma, *Vb* vascular bundle, *En* endodermis

The surface of the stem exhibited a densely pubescent indumentum (Fig. 3a, c, e, f). It mainly consisted of long multicellular trichomes (Fig. 3c), among which shorter 8–9-celled non-glandular trichomes were found (Fig. 3b–d). Their basal cells were much wider than the apical ones. Their erect trichomes were inclined towards the surface of the organ (Fig. 3b–d). Furthermore, there were sporadic biseriate glandular trichomes, the same as those on the surface of the bracts (Fig. 3f).

The stem had a pentagonal shape, which was visible in the cross section (Fig. 3e, f). There was one layer of epidermal cells with a thick cuticle. Below, there was a multilayered cortex, which most often consisted of one layer of collenchyma and several layers of parenchymatous cells (Fig. 3e–g). The collenchyma in the stem corners was much thicker and consisted mostly of 5–6 layers of cells (Fig. 3e, f). Vascular bundles of various sizes were embedded in the stele parenchyma and surrounded by a sheath of sclerenchyma (Fig. 3e–g). Individual reservoirs (ducts) with yellowish secretion were located in the cortex parenchyma outside the endodermis. The ducts were mainly located above the phloem and the outer part of the sclerenchyma sheath (Fig. 3g, h). The ducts were surrounded by a single layer of epithelium consisting of 5–6 cells. Their diameter was approximately 3.5 μm.

### Histochemistry of stem secretory ducts

The histochemical assays and fluorescence microscopy studies showed that the secretion of the stem secretory ducts contained different secondary metabolites: lipids, essential oil, terpenoids and phenolic compounds (tannins and flavonoids).

In control samples submerged in water, the secretion in the ducts was yellowish (Fig. 3g, h). The presence of lipids in the secretory product was detected after application of Sudan IV and Sudan Red B, which stained total lipids orange or reddish, respectively (Fig. 3i, j). After the Nile Blue treatments, acid lipids were visualised by blue colour (Fig. 3k). The presence of lipid substances was also confirmed by green autofluorescence under the FITC filter set (Fig. 3l). Essential oil contained in the secretion was stained red upon the Neutral Red treatment (Fig. 3m). We also showed the presence of tannins, which were characterised by light brown colour when stained with potassium dichromate (Fig. 3n). We observed yellowish secondary fluorescence after using aluminium trichloride and magnesium acetate as fluorochromes, confirming the presence of flavonoids (Fig. 3o, p). The presence of terpenes containing steroids was indicated by intense yellowish fluorescence under UV light (Fig. 3q).

The reactions were negative or similar to the control after application of the other histochemical assays based on the use of periodic acid-Schiff’s reagent, Ruthenium Red, Nadi reagent, concentrated sulphuric acid and ferric trichloride.

### Morphology and anatomy of leaves

**Fig. 4:**
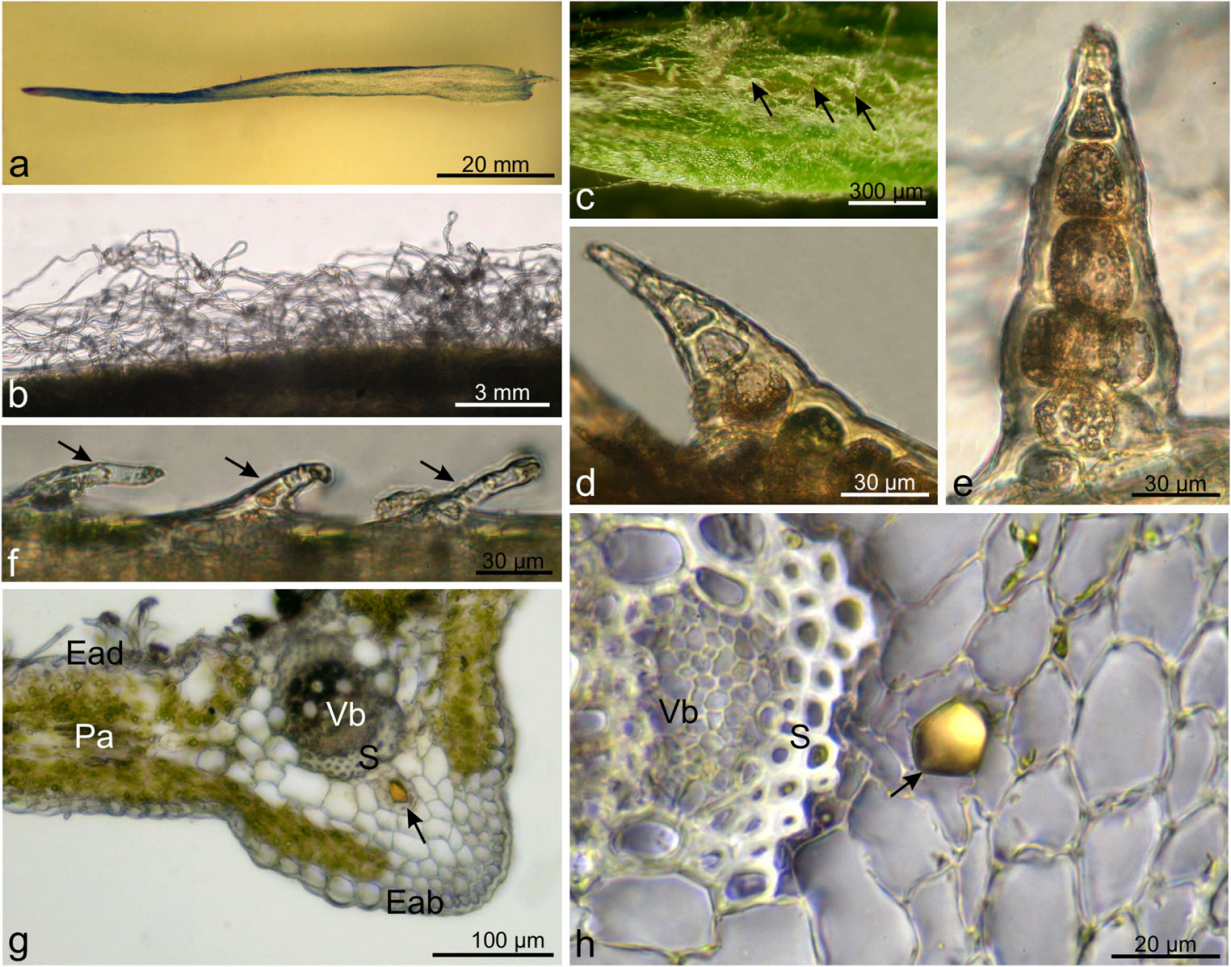
Microstructure of *C. cyanus* leaves. **a** Adaxial leaf surface covered with a white densely pubescent indumentum. **b** Long, white non-glandular trichomes on the adaxial leaf surface. **c** Portion of the abaxial leaf surface with short non-glandular trichomes (arrows) on the midrib. **d**, **e** Live non-glandular trichomes with thick-walled cells on the abaxial leaf surface. **f** Short non-glandular trichomes (*arrows*) on the leaf margin. **g** Portion of a transverse section through the leaf with a visible vascular bundle and secretory duct (*arrow*) in the midrib. **h** Secretory duct with yellow secretion (*arrow*) above the sclerenchyma sheath of vascular bundle. *Vb* vascular bundles, *S* sclerenchyma sheath, *Pa* mesophyll, *Ead* adaxial epidermis, *Eab* abaxial epidermis

*Centaurea cyanus* leaves are lanceolate with a pointed apical part (Fig. 4a). Similar to the bract and stem surfaces, the adaxial surface of the leaf was covered by long non-glandular trichomes forming a densely pubescent indumentum (Fig. 4b). These uniseriate and multicellular trichomes had a long thread-like terminal cell (not shown). On the abaxial leaf surface, we observed two types of non-glandular trichomes. The first type was represented by sparse long uniseriate and multicellular non-glandular trichomes. The second type comprised much shorter (approximately 107 μm long) pointed non-glandular trichomes composed of several (6–8) thick-walled cells with an enlarged double-row base, similar to those found on the stem. They were located on the midrib surface (Fig. 4c-e). The cells of these trichomes exhibited light brown granular content (Fig. 4d, e). Additionally, curved, live non-glandular trichomes composed of 4–6 cells with a length of approx. 40 μm were visible on the leaf margins (Fig. 4f).

The cuticle-covered cells on both epidermis (upper and lower) surfaces had thick outer walls. The next layer of tissue consisted of cylindrical, elongated, chloroplast-rich cells forming the palisade parenchyma. Below, there was spongy parenchyma with irregularly shaped cells. Vascular bundles were arranged in the middle part of the parenchyma. The bundles from the top and bottom were delimited by a sclerenchymatous sheath (Fig. 4g, h). The largest vascular bundle was located in the central region of the leaf blade. A single secretory duct, similar to those found in the stem, was visible in the parenchyma below the lower layer of the sclerenchyma sheath surrounding the main bundle (Fig. 4g, h).

### Ultrastructure of stem secretory ducts

**Fig. 5:**
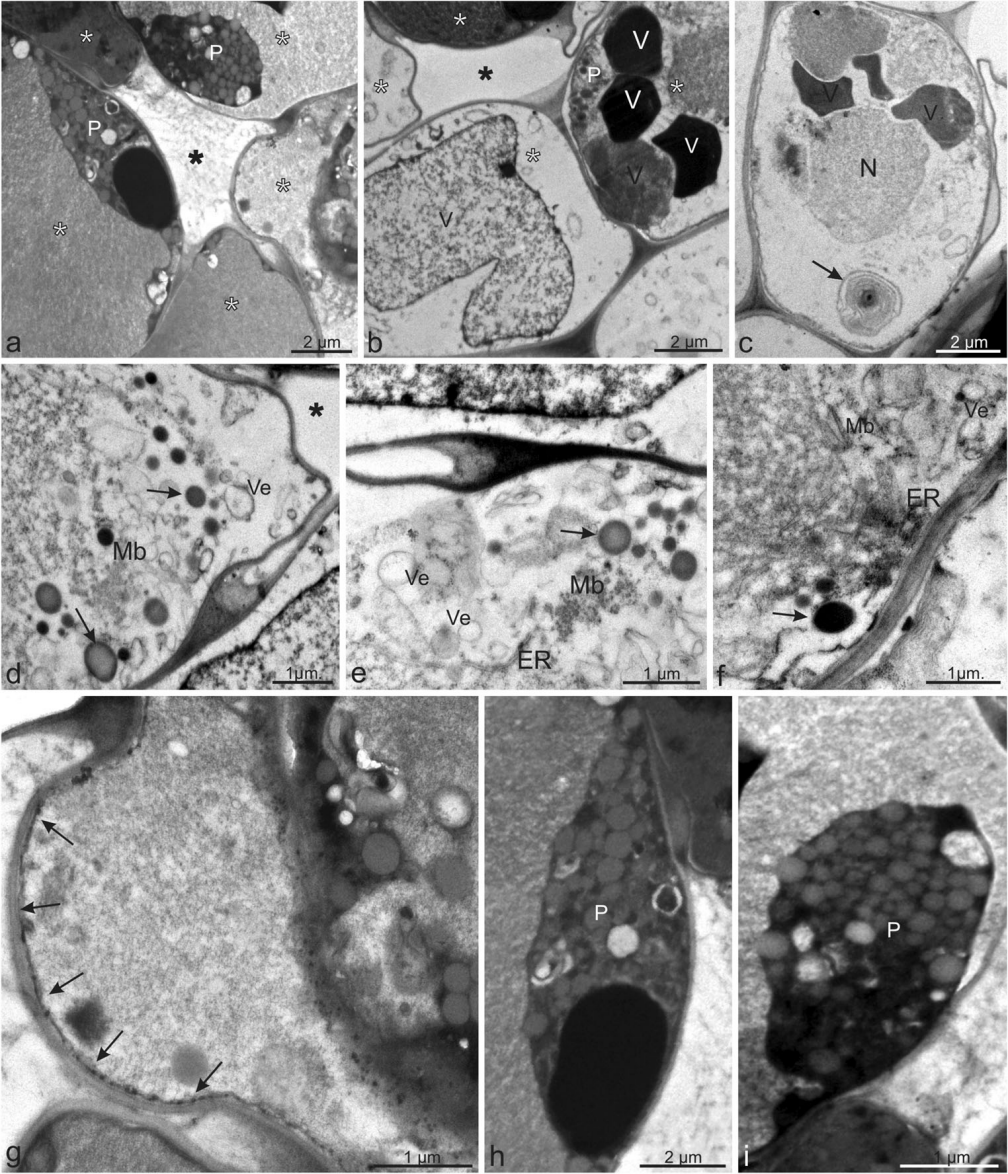
Ultrastructure of the secretory ducts in the *C. cyanus* stem. **a**, **b** Secretory ducts (*black asterisks*) surrounded by a layer of epithelial cells (*white asterisks*). Note that every glandular cell was in different secretory phases. **a**, **c** Younger cells of the epithelium had a visible nucleus and plastids. **b** Older epithelial cells had cytoplasm limited to the peripheral region and the cell interior filled with a large central vacuole containing flocculent material with moderate electron density. **c** Note myelin-like bodies (*arrow*) in the cytoplasm. **d**–**f** In the older cells, a visible degraded protoplast with numerous large vesicles with different degrees of osmophilicity accumulated along the cell wall. Note the small lipid bodies (*arrows*) and multivesicular or membranous bodies. **g** Long chains of granular lipophilic material (*arrows*) visible between the cytoplasm and the cell wall. **h**, **i** In the cytoplasm, visible dark elaioplasts enriched with lipidic substances. *N* nucleus, *P* plastids, *ER* endoplasmic reticulum, *V* vacuoles, *Ve* vesicles, *Mb* multivesicular and membranous bodies

The ultrastructure of epithelial cells located around the secretory duct in the stem was analysed. The duct is a tubular structure lined by one layer of epithelial secretory cells (Fig. 5a, b). The secretory cells differed from the surrounding parenchyma cells—they were smaller and had abundant organelles. In turn, the parenchyma cells were substantially bigger and were filled with a huge vacuole. It was evident that every secretory cell was in another secretory phase (Fig. 5a, b). Older secretory cells had released a secretion and were in the postsecretory stage. In these cells, the cytoplasm was limited to the peripheral region, and the cell interior was filled with a large central vacuole containing flocculent material with moderate electron density (Fig. 5b). The cell protoplast was largely degraded, and numerous vesicles with different degrees of osmophilicity were accumulated mainly along the cell wall (Fig. 5d–f). These secretory cells were characterised by the presence of multivesicular or membranous bodies, probably filled with lipophilic compounds, as the secretion stained with e.g. Sudan also revealed the presence of these compounds (Fig. 5d–f). In turn, younger cells that were in the secretory stage had a visible nucleus and plastids (Fig. 5a, c). Moreover, there were small vacuoles filled with a strongly osmiophilic non-soluble fraction of a substance representing phenolic compounds, which was confirmed by the histochemical tests (Fig. 5c). The cytoplasm also contained myelin-like bodies probably derived from the smooth endoplasmic reticulum or formed by the disruption of plastid membranes (Fig. 5c). Long chains of granular lipophilic material were present between the cytoplasm and the cell wall (Fig. 5g). The vesicles with various substances transported them through the cytoplasm and fused with the cell membrane via endocytosis. Dark elaioplasts enriched with lipophilic compounds were observed in the cytoplasm (Fig. 5h, i). No plasmodesmata were observed in the walls of the epithelial cells of the secretory ducts. The microscopic analyses suggested that the ducts developed through separation (middle lamella detachment) of the walls of the central cells, which were pushed outward. Furthermore, the presence of epithelial cells surrounding the duct confirmed their schizogenous origin.

## Discussion

Recently, *Centaurea* has become the subject of intensive research, as several species from this genus have medicinal properties. Many studies on phytochemical characterisation have been conducted (e.g. Karamenderes et al. 2007; Küpeli Akkol et al. 2009; Aktumsek et al. 2011; Luković et al. 2013), yet the structure of these species has still been poorly described. Although chemical analyses and molecular data have greatly contributed to establishment of the species phylogeny, macro- and micromorphological features are still an irreplaceable source of information for this type of study.

In the *C. cyanus* disc flowers, we observed densely distributed papillae at the top of the corolla lobes. Similar location of papillae has been reported in flowers of other Asteraceae species, e.g. representatives of the genera *Petasites* (Haratym and Weryszko–Chmielewska 2012), *Lessingianthus* (Angulo and Dematteis 2014), *Gazania* and *Cirsium* (personal observations). The presence of papillae in the apical zone of corolla lobes seems to be a characteristic trait of many Asteraceae species.

The surfaces of ray flower petals in *C. cyanus* (Cardueae) had a crested cuticular pattern, similar to that described earlier by other authors in the tribes Arctotideae, Calenduleae, Cichorieae, Inuleae and Mutisieae (Baagøe 1977, 1978; Hansen 1991; Koch et al. 2013). For the first time, our results provide information about such cuticular sculpture in the ray flowers in *C. cyanus*. A longitudinal cuticular pattern was detected on the stylar and filament hairs in the *C. cyanus* flowers. Our observations are in agreement with the data on the cuticular pattern on stylar hairs reported by Erbar and Leins (2015) for the Cardueae tribe. The longitudinal pattern on stylar hairs is observed in Asteraceae relatively frequently; for instance, Erbar and Leins (2015) have described this trait in 17 of all the 44 tribes.

In our study, we have demonstrated that *C. cyanus* stamens were equipped with a mechanism that facilitated the release of pollen grains. In our opinion, the spirally twisted structures present at the bottom of the anthers might be involved in their shrinkage and cracking, as the anther tube shrank in response to an insect touch. In turn, the numerous concentrically arranged hair-like structures located on the filaments probably protect nectar against water flow and thus nectar dilution and/or drying out.

The morphology and distribution of trichomes are regarded as a representative feature in the Asteraceae family (e.g. Ciccarelli et al. 2007; Hayat et al. 2009; Rahiminejad et al. 2010; de Andrade et al., 2014a, b). The trichomes described in *C. cyanus* in this study represent four types of non-glandular trichomes (one type of longer hairs and 3 types of shorter trichomes) and one type of glandular trichomes (biseriate trichomes). All these trichome types were located on the stems and bracts, whereas only long and short non-glandular hairs were visible on the leaves. The density of the trichomes varied. In turn, Chiru et al. (2013) observed three types of non-glandular trichomes in *C. cyanus*. While, Luković et al. (2013) described two types of these trichomes in *Centaurea sadleriana*. We have demonstrated that the glandular trichomes found on the epidermal surface of *C. cyanus* bracts have 6–7 tiers. Werker et al. (1994) observed glandular trichomes on the surface of *Artemisia dracunculus* with the same number of cell layers. Biseriate trichomes differing in the number of cells and size from those present in *C. cyanus* have been described in many other species, e.g. *Artemisia annua* (Duke and Paul 1993), *Chamomilla recutita* (Sulborska 2011), *Helichrysum aureonitens* (Afolayan and Meyer 1995), *Inula helenium* (Sulborska 2013), *Sigesbeckia jorullensis* (Heinrich et al. 2002) and *Stevia rebaudiana* (Bondarev et al. 2010).

In the abaxial epidermal cells of the bracts in *C. cyanus*, we observed numerous prismatic calcium oxalate crystals. Crystals are present in different plant organs including leaves, stems, roots and fruits as well as various floral organs such as ovaries, anthers, petals, sepals or bracts, and nectaries (Dane et al. 2000, Meric, 2009a, b; Jacobs et al. 2010; Horner 2012). Several studies have shown their variety in the Asteraceae family. Some species contain raphides (Kartal 2016), druses (Nwosu et al. 2013), styloids (Meric, 2009a, b) and prismatics (Meric 2009b). The investigations conducted by Kartal (2016) revealed that styloids and prismatics are the most common forms of crystals found in the members of the Cardueae tribe. The present study examined the morphology and location of CaO_x_ crystals in *C. cyanus*, which also belongs to the Cardueae tribe. Their prismatic shape was similar to that of crystals found in other species of *Centaurea*—*C. iberica* and *C. salonitana* (Kartal 2016). Although the distribution and shapes of calcium oxalate crystals can be affected by biological, chemical and physical conditions, their formation is believed to be controlled genetically; thus, they are species specific (Prychid and Rudall 1999; Franceschi and Nakata 2005). Therefore, their types as well as presence or absence can be used as taxonomic characters (Kuo–Huang et al. 2007; Horner et al. 2009, 2012). Their possible roles can include regulation of calcium concentration, detoxification of heavy metals or oxalic acid, light gathering and reflection, protection against herbivores, and strengthening of tissues (Franceschi and Nakata 2005; Kuo–Huang et al. 2007; Cote and Gibernau 2012). The results of the latest research have shown that these crystals can also be an important internal source of CO_2_ used by plants for the so-called alarm photosynthesis (Tooulakou et al. 2016).

The secretory ducts of *C. cyanus* were formed schizogenously: the schizogenous corner-space was created by gradual separation of neighbouring cell walls anticlinal to the reservoir along the middle lamella. The development of this structure in the *C. cyanus* stems and leaves agrees with the observation made in other species from the Asteraceae family such as *Arnica montana*, *Flourensia campestris*, *F. oolepis*, *Matricaria chamomilla* or *Rhaponticum carthamoides* (Łotocka and Geszprych 2004; Andreucci 2008; Silva et al. 2015; Kromer et al. 2016). We found secretory ducts in the stems of *C. cyanus* only in the region of endodermis opposite the phloem. However, we did not detect medullary canals, which were found in *Centaurea* by Metcalfe and Chalk (1972). The anatomy of these internal secretory structures is similar to that of other species in the same family, e.g. *Inula helenium* (Sulborska 2007)*.* Moreover, similar to *C. cyanus*, also in *C. sadleriana* (Luković et al. 2013), only a single canal was present between parenchymatic cells located close to the phloem, while usually two cavities were detected in *I. helenium*—one on each side of the bundle. In *I. helenium*, sometimes even four cavities accompanied a single vascular bundle, i.e. two on each of its sides. The presence of only one secretory reservoir was rare. However, in another species from the Asteraceae family—*Petasites hybridus*, secretory ducts were observed in the cross section of scaly leaves in the same position as those in the *C. cyanus* (Haratym and Weryszko–Chmielewska 2012). Since they are located close to the phloem in various species, secretory ducts may aid the transfer of organic material by sieve tubes (Williams 1954). Moreover, these ducts in the Cardueae tribe can help to distinguish between single species. Fritz and Saukel (2011) revealed that the ratio between the diameter of the duct and the size of surrounding parenchyma cells is an important feature in identification of species in a given genus.

The results of the histochemical assays and fluorescence microscopy revealed that the material produced by the epithelium cells of the secretory ducts of *Centaurea cyanus* have a complex nature. Most of the substances produced by the *C. cyanus* are lipophilic and lipidic, which was indicated by the positive reactions with Sudan IV, Sudan Red and Nile Blue and by the autofluorescence observed with the use of the FITC filter. Production of lipids by the epithelium cells of the secretory ducts was also found in *Matricaria chamomilla*, *Santolina ligustica*, and species from the *Ophryosporus* genus (Pagni et al. 2003; Andreucci et al. 2008; Plos et al. 2011), which belong to the same family as the *C. cyanus*. The presence of essential oil was confirmed by the reaction with Neutral Red. Numerous species in the Asteraceae family produce these metabolites as well, e.g. *Matricaria chamomilla* (Andreucci et al., 2008), *Pteronia incana* (Hulley et al. 2010), and different species of *Baccharis* (Budel et al. 2012). Moreover, the reaction with potassium dichromate facilitated detection of tannins in the *C. cyanus* secretion. As in the present study, the production of flavonoids by *C. cyanus* organs was also reported by Litvinenko and Bubenchikova (2007). Both tannins and flavonoids are described as phenolic compounds; they have gastroprotective properties and an ability to inhibit oxidative processes and aid the control of gall bladder (Pirvu et al. 2012). Furthermore, flavonoids in plants are responsible for the colour and aroma of flowers and fruits, which is important for attracting pollinators and seed dispersal. These compounds protect plants against various biotic and abiotic stresses and act as a unique UV filter. In addition, they are used as signal molecules, allelopathic compounds, phytoalexins, detoxification agents and repellents of pathogenic microorganism and other pests and play a role in frost and drought resistance (Jurzitza 1987; Harborne 1988; Amalesh et al. 2011). In our study, the presence of terpenes containing steroids in the ducts was revealed with the use of antimony trichloride. Fernandez et al. (1995) found that loliolide was produced in *C. cyanus* organs. This terpene compound has many biological properties, e.g. antibacterial, anti-cancer, antifungal and antioxidant activity. Furthermore, plants containing loliolide are used in alternative medicine in the treatment of diabetes and depression (Grabarczyk et al. 2015). In other studies, this compound was present in other Asteraceae species such as *Artemisia suksdorfii* or *Helianthus tuberosus* (Ahmed et al. 2004; Pan et al. 2009). The histochemical assays of the *C. cyanus* secretory ducts did not reveal the presence of pectic-like substances. Similar observations were made in the research of reservoirs found in *Matricaria chamomilla* or *Santolina ligustica* (Pagni et al. 2003; Andreucci et al. 2008).

The ducts found in the *C. cyanus* organs were delimited by a single epithelial layer mostly consisting of five secretory cells. A comparison of the number of layers of epithelial cells between secretory structures in other species showed a strong resemblance to *Tagetes patula*, in which a uniseriate epithelium was detected as well (Poli et al. 1995). In the Asteraceae family, there are species having cavities with a biseriate epithelium (*Conyza canadensis*) (Lersten and Curtis 1987) or several layers of glandular cells (*Tagetes minuta*) (Simon et al. 2002).

In *C. cyanus*, numerous vesicles containing a heterogeneous secretory substance were detected in close vicinity of the epithelial cell walls. Similar structures were also described in epithelial cells of other Asteraceae representatives, e.g. *Inula helenium* (Sulborska 2007) and *Rhaponticum carthamoides* (Łotocka and Geszprych 2004). Myelin-like figures were visible in the secretory cells in the cytoplasm as well. They had the same appearance as those found in the *Inula helenium* nectary (Sulborska and Weryszko-Chmielewska 2007). Elaioplasts, which stored fats, were found in the structure of the epithelial cells. This type of plastids was described in *Grindelia pulchella* (Asteraceae) glandular trichomes and ducts by Bartoli et al. (2011).

## Conclusions

The aboveground organs of *Centaurea cyanus* exhibited four types of non-glandular trichomes, which may have taxonomic value and deter herbivores. Taxonomic relevance can also be ascribed to the papillae present on the petal lobes, the cuticular pattern observed on the petals of ray flowers as well as stylar and stamen hairs, and the prismatic deposits of calcium oxalate crystals in the bracts. Moreover, the presence of the hair-like structures on the *C. cyanus* flowers indicated their possible involvement in protection of nectar against dilution and/or drying out (trichomes on the surface of the stamens) and their role in secondary pollen presentation (trichomes and papillae on the pistil surface, which play an important role in flower pollination).

Two main secretory structure types, i.e. biseriate glandular trichomes and ducts, were found in the *C. cyanus* organs. The histochemical assays indicated the presence of various compounds such as essential oil, flavonoids, lipids, tannins and terpenes containing steroids and revealed a heterogeneous nature of the *C. cyanus* secretion produced by the ducts. The secretory ducts located in the stems were formed schizogenously. The epithelial cells of the same duct were in different stages of development. In the epithelial cells, and strongly osmiophilic insoluble phenolic material was stored in the vacuoles, whereas elaioplasts and vesicles contained, moderately osmiophilic insoluble lipidic material.
